# Characteristics and contributing factors related to sports injuries in young volleyball players

**DOI:** 10.1186/1756-0500-6-415

**Published:** 2013-10-14

**Authors:** Franciele Marques Vanderlei, Fabio Nascimento Bastos, Gustavo Yuki Cantalejo Tsutsumi, Luiz Carlos Marques Vanderlei, Jayme Netto, Carlos Marcelo Pastre

**Affiliations:** 1 Master in physiotherapy by Univ Estadual Paulista, Faculdade de Ciências e Tecnologia, Presidente Prudente, SP, Brazil, Roberto Simonsen, 305, Presidente Prudente, SP CEP 19060-900, Brazil; 2 Post graduate Program in Experimental Pathology, Univ Estadual de Londrina, Londrina PR, Brazil; 3 Especializing in Sports Physiotherapy, UNESP – Univ Estadual Paulista. Faculdade de Ciências e Tecnologia, Presidente Prudente SP, Brazil; 4 Department of Physiotherapy, UNESP – Univ Estadual Paulista. Faculdade de Ciências e Tecnologia, Presidente Prudente SP, Brazil

**Keywords:** Trauma in athletes, Risk factors, Volleyball

## Abstract

**Background:**

The participation of young in volleyball is becoming increasingly common, and this increased involvement raises concerns about the risk of installation of sports injuries. Therefore, the objectives the study were identify the characteristics of sports injuries in young volleyball players and associate anthropometric and training variables with contributing factors for injuries.

**Methods:**

A total of 522 volleyball players participating in the High School Olympic Games of the State of São Paulo (Brazil) were interviewed. A reported condition inquiry was used to gather information on injuries, such as anatomic site affected, mechanism and moment of injury, as well as personal and training data. The level of significance was set at 5%.

**Results:**

A 19% frequency of injuries was found. Higher age, weight, height, body mass index and training duration values were associated with the occurrence of injuries. The most affected anatomic site was the ankle/foot complex (45 injuries, 36.3%). Direct contact and contactless mechanisms were the main causes of injuries (61 injuries; 49.2% and 48 injuries; 38.7%, respectively). Training was the moment in which most injuries occurred (93 injuries; 75%), independently of personal and training characteristics.

**Conclusion:**

Injuries affected the ankle/foot complex with a greater frequency. Direct contact and contactless mechanisms were the most frequently reported and injuries occurred mainly during training sessions. Personal and training characteristics were contributing factors for the occurrence of injuries.

## Background

Volleyball is one of the most popular sports in the world [1] and is practiced by approximately 800 million people [2] with diverse characteristics, including different age groups. A large number of youths are involved in this sport and, as in any other sport, are exposed to injuries.

Sports injuries are related to diverse factors, such as gender, duration of training and competition as well as anthropometric and physical characteristics [1,3]. In youths, there is the additional concern regarding the integrity of the bodily structures of the practitioners of sports [4,5], as musculoskeletal immaturity in this population may be a risk factor for injuries [6]. Moreover, constant exposure to repetitive motor actions and excessive joint loads leads to a greater risk of injury.

In the literature, studies about major sports injuries in young volleyball players showed some injuries predominate. Carazzato et al. [7] identified the prevalence of knee injuries (26.74%), ankle (19.52%), spine (13.44%), hand (13.3%) and shoulder (7.9%). While Agel et al. [8] found that 55% of the injuries occurred in the lower limbs, with predominance to ligamentous sprains ankle, and approximately 20% in the upper limbs.

In Brazil, despite the observed increase in recent years of the number of young involved in sports, there is a lack of adequate medical facilities and efficient surveillance systems of injuries for young athletes unlike what is observed in high-level competition [9]. So given the vulnerability of young athletes regarding sports injuries, it is understood that more studies are needed on the national level, which may provide better control over the occurrence of these injuries.

There is a close relationship between sports practice and sports injury, however, there is shortage of Brazilian research about sports injuries in young volleyball athletes. The investigation of injuries in young volleyball players may facilitate the establishment of better, consistent preventive strategies [10]. This population is in the process of structural and motor development [11] and early injuries to the locomotor apparatus can affect future motor actions and even jeopardize the continuity in the athletic career [12]. Thus, there is a need to investigate and associate the different risk factors of sports injuries in order to assist in the determination of specific strategies regarding injury prevention and health maintenance among volleyball players.

The aims of the present study were to identify the characteristics of sports injuries in young volleyball players through the administration of a reported condition inquiry and to associate anthropometric (age, weight, height and body mass index) and training (duration of sports practice) as contributing factors for injuries.

## Method

A total of 522 volleyball players participating in the High School Olympic Games of the State of São Paulo (Brazil) were approached (249 females and 273 males; mean age: 14.96 ± 1.67 years; body mass: 60.88 ± 12.43 kg; height: 1.69 ± 0.11 m; body mass index (BMI): 21.08 ± 2.81 kg · m^-2^; and duration of training: 3.24 ± 1.68 years). The volunteers were classified as young (aged 12 to 18 years), based on the classification determined in the Brazilian Child and Adolescent Statute established in Brazil in 1990 [13].The volunteers and their respective parents/guardians were informed as to the purpose of the study, agreed to participate and signed a statement of informed consent. The study received approval from the Human Research Ethics Committee of the *Universidade Estadual Paulista/Faculdade de Ciências e Tecnologia* (Brazil) under protocol number 221/2007.

A retrospective study was carried out. The data were collected through individual interviews using a reported condition inquiry addressing the occurrence of injury and its characteristics in the current season (previous 12 months of training and/or competition). The volunteers were approached either prior to or following training sessions in order not to interfere in the normal dynamics and routine of the sport. The data were collected using the Referred Condition Inquiry in the period from competition (during one month).

The reported condition inquiry is widely used for the acquisition of information on general health status in a specific population due mainly to its applicability and objectivity [14,15]. A pilot study was first conducted to adjust the data acquisition procedures and test the inquiry on a population with similar characteristics to those of the present study, which confirmed the full possibility of use and fit in the proposed methodological design.

The interviews were carried out by single interviewer familiarized with the instrument. Following the presentation of the objectives and legal terms for participation in the study, the volleyball players responded to the questions posed by the researcher, who was also responsible for recording the answers on the inquiry in an individual fashion. Pastre *et al.*[14] suggests this procedure based on different degrees of understanding regarding the annotation of answers on the part of interviewees. The forms were then numbered in order to facilitate the recording of the information and the data were recorded on computational spreadsheets for organization, systematization and analysis.

The inquiry contained personal data, such as gender, age, reported weight, reported height, duration of training in years and BMI, which was subsequently calculated using the following formula: BMI = body mass · height^-2^. For the acquisition of information on injuries, the inquiry posed questions on the anatomic site affected, injury mechanism and moment of injury (Additional file 1: Appendix). An illustration of the human body was shown to the volunteer in order to facilitate the identification of the anatomic site of the injury and the volunteer marked the region of the body referring to the sensation of pain or musculoskeletal discomfort. The determination of the injury mechanism consisted of the volunteer’s perception regarding the contact or exact action performed when signs and symptoms of an acute episode emerged and/or the type of activity in which such manifestations were accentuated. This variable was divided into direct contact, contactless and overuse. The moment of occurrence of the injury was analyzed based on the specific phase of training or competition.

For the present study, sports injury was defined as any impairment of the musculoskeletal system with signs and symptoms stemming from the practice of the sport in either the training or competition phase that compromised normal training in terms of form, duration, intensity or frequency, as already used in previous studies [14,15].

To facilitate the analysis and presentation of the results, the variables were subdivided into categories based on the most expressive clusters of results without affecting the essence of their origin or the conclusions of the study. Regarding anatomic site of pain or discomfort, the questionnaire listed 20 bodily regions, which were grouped in the following segments: trunk, shoulder, wrist/hand, thigh/leg and ankle/foot.

The following three injury mechanisms were considered: i) injury due to direct contact caused by a single traumatic incident, such as collision with opponent and fall [8,16]; ii) contactless injuries stemming from aspects inherent to the sport itself, such as short and long-distance runs, rapid changes in movement, jumps and landing [8,16]; and iii) injuries due to overuse, presenting in the form of chronic injury stemming from repetitive effort of the musculoskeletal system over time without allowing adequate recovery [17,18].

Descriptive statistics were used for the analysis of the profile of the population and description of the variables. The results were expressed as mean and standard deviation values, percentages and absolute numbers. The odds ratio (OR) test with a 95% confidence interval (CI) was used initially to determine the existence of an association between gender and the presence/absence of injury. As the results revealed no significant differences, the analyses were performed without gender distinctions. The Kolmogorov-Smirnov was used to test the normality of the data.

For comparison of factors associated with the occurrence of injury was applied the Student’s t-test for non-paired data in cases of normal distribution (height) and the Mann–Whitney test for cases in which normal distribution was not found (age, body mass, BMI and duration of training). For variables that were statistically different (p < 0.05) in relation to occurrence of injury was established cutoff points through the range of 95%. After the selection of cutoff points was applied test OR and 95% confidence interval to determine whether there were statistical differences between individuals who were injured below and above the cutoff preset. The variables that showed differences were analyzed using the Goodman’s test for contrasts between and within multinomial populations to test associations between anatomic site, injury mechanism and moment of injury according to the cutoff of the group of variables to be analyzed. The statistical analyses were conducted using the SPSS program (SPSS, USA, Version 17), with the significance level set at p < 0.05.

## Results

Among the 522 interviewees, 104 (19.92%) reported a total of 124 injuries, with a risk of 0.237 injuries/participant and 1.19 injuries/injured participant. The frequency distribution of injuries according to gender did not achieve a statistically significant difference (OR: 1.40; 95% CI: 0.93–2.09).

Table 1 displays the measures of central tendency and variability for the variables age, body mass, height, BMI and duration of practice according to the occurrence of injury. For all these variables, significant differences were found between athletes who suffered injuries and those who did not suffer injuries.

**Table 1:** Mean and standard deviation values for age, body mass, height, body mass index (BMI) and duration of practice according to occurrence of injury

**Variable**	**Injury**	
	**Presence (n = 104)**	**Absence (n = 418)**
**Age (years)**	15.55 ± 1.47*	14.78 ± 1.69
**Body mass (kg)**	66.63 ± 13.36*	59.09 ± 11.58
**Height (m)**	1.74 ± 0.12*	1.67 ± 0.10
**BMI (kg · m**^**-2**^**)**	21.73 ± 2.57*	20.88 ± 2.85
**Duration of practice (years)**	3.62 ± 1.66*	3.13 ± 1.67

Note: *Statistically significant difference in relation to absence of injury (p < 0.05). The Student’s t-test for independent samples was used to compare means in two groups for height. The Mann–Whitney test was used to compare medians in two groups for age, body mass, body mass index and duration of practice.

Differences were found between athletes who suffered injuries and those who did not suffer injuries for the variables age, body mass, height, BMI and duration of practice (p < 0.05, for all differences). The OR test with a 95% confidence interval was performed to determine the risk factor among the participants having suffered injuries.

Duration of practice was the only variable for which no statistically significance difference was found (OR: 1.48; CI 95%: 0.97–2.26) when comparing athletes who practiced more than 3.62 years and those with less practice duration. The other variables exhibited significant differences and were separated by the lower or upper cutoff point of the lower limit of 95% confidence interval. Older athletes (more than 15.55 years) had a greater risk of injury in comparison to younger athletes (OR: 2.84; CI 95%: 1.84–4.39). Heavier athletes (more than 66.63 kg) had greater risk of injury in comparison to lighter athletes (OR: 2.90; CI 95%: 1.92–4.38). Taller athletes (more than 1.74 m) had greater risk of injury in comparison to shorter athletes (OR: 2.76; CI 95%: 1.83–4.17). Participants with a BMI higher than 21.73 kg · m^-2^ had greater risk of injury in comparison to those with a lower BMI (OR: 1.76; CI 95%: 1.17–2.64). Therefore, these variables were tested for associations with the characteristics of the injuries (anatomic site, mechanism and moment of injury).

Table 2 shows that older and heavier athletes had a greater frequency of injury in the ankle/foot in comparison to the shoulder, thigh/leg and trunk. The data demonstrate that taller volunteers and those with a higher BMI reported a greater frequency of injury in the ankle/foot in comparison to thigh/leg and trunk. No significant differences were found among the personal and training variables in relation to anatomic site.

**Table 2:** Absolute and relative distribution of age, body mass, height and body mass index (BMI) according to anatomic site affected

**Variables**	**Characteristic**	**Anatomic site**					
		**Trunk**	**Shoulder**	**Wrist/hand**	**Thigh/leg**	**Knee**	**Foot/ankle**
**Age**	**Younger**^**1**^	5 (13.9)	2 (5.5)	9 (25.0)	5 (13.9)	5 (13.9)	10 (27.8)
	**Older**^**2**^	7 (7.9)*	11 (12.5)*	16 (18.2)	5 (5.7)*	14 (15.9)	35 (39.8)
**Body mass**	**Lighter**^**3**^	6 (11.1)	6 (11.1)	15 (27.8)	4 (7.4)	8 (14.8)	15 (27.8)
	**Heavier**^**4**^	6 (8.6)*	7 (10.0)*	10 (14.3)	6 (8.6)*	11 (15.7)	30 (42.8)
**Height**	**Shorter**^**5**^	6 (11.4)	5 (9.4)	11 (20.7)	4 (7.5)	9 (17.0)	18 (34.0)
	**Taller**^**6**^	6 (8.5)*	8 (11.2)	14 (19.7)	6 (8.5)*	10 (14.1)	27 (38.0)
**BMI**	**Lower BMI**^**7**^	4 (7.4)	5 (9.3)	11 (20.4)	6 (11.1)	8 (14.8)	20 (37.0)
	**Higher BMI**^**8**^	8 (11.4)	8 (11.4)	14 (20.0)	4 (5.8)*	11 (15.7)	25 (35.7)

^*1*^*cutoff point of lower bound of confidence interval (CI) (< 15.29 years) obtained from participants for age variable;*^*2*^*cutoff point for upper bound of CI (≥15.29 years) obtained from participants for age variable;*^*3*^*cutoff point of lower bound of CI (< 64.28 kg) obtained from participants for body mass variable;*^*4*^*cutoff point for upper bound of CI (≥64.28 kg) obtained from participants for body mass variable;*^*5*^*cutoff point of lower bound of CI (< 1.72 m) obtained from participants for height variable;*^*6*^*cutoff point for upper bound of CI (≥1.72 m) obtained from participants for height variable;*^*7*^*cutoff point of lower bound of CI (< 21.28 kg · m*^*-2*^*) obtained from participants for BMI variable;*^*8*^*cutoff point for upper bound of CI (≥21.28 kg · m*^*-2*^) obtained from participants for BMI variable; *Significant difference in relation to foot/ankle (p < 0.05).

There was no significant difference among the younger athletes regarding the mechanism of injury. For the other variables, direct contact and contactless mechanisms were greater causal factors of injury (except in taller athletes) in comparison to overuse. No significant differences were found among the personal and training variables in relation to the injury mechanism (Table 3).

**Table 3:** Absolute and relative distribution of age, body mass, height and body mass index (BMI) according to mechanism of injury

**Variables**	**Characteristic**	**Mechanism of injury**		
		**Direct contact**	**Contactless**	**Overuse**
**Age**	**Younger**^**1**^	19 (52.8)	11 (30.5)	6 (16.7)
	**Older**^**2**^	42 (47.8)*	37 (42.0)*	9 (10.2)
**Body mass**	**Lighter**^**3**^	27 (50.0)*	21 (38.9)*	6 (11.1)
	**Heavier**^**4**^	34 (48.6)*	27 (38.6)*	9 (12.8)
**Height**	**Short**^**5**^	27 (50.9)*	22 (41.6)*	4 (7.5)
	**Tall**^**6**^	34 (47.9)*	26 (36.6)	11 (15.5)
**BMI**	**Less BMI**^**7**^	30 (55.6)*	19 (35.2)*	5 (9.2)
	**More BMI**^**8**^	31 (44.2)*	29 (41.5)*	10 (14.3)

^*1*^*cutoff point of lower bound of confidence interval (CI) (< 15.29 years) obtained from participants for age variable;*^*2*^*cutoff point for upper bound of CI (≥15.29 years) obtained from participants for age variable;*^*3*^*cutoff point of lower bound of CI (< 64.28 kg) obtained from participants for body mass variable;*^*4*^*cutoff point for upper bound of CI (≥64.28 kg) obtained from participants for body mass variable;*^*5*^*cutoff point of lower bound of CI (< 1.72 m) obtained from participants for height variable;*^*6*^*cutoff point for upper bound of CI (≥1.72 m) obtained from participants for height variable;*^*7*^*cutoff point of lower bound of CI (< 21.28 kg · m*^*-2*^*) obtained from participants for BMI variable;*^*8*^*cutoff point for upper bound of CI (≥21.28 kg · m*^*-2*^) obtained from participants for BMI variable; *Significant difference in relation overuse (p < 0.05).

Table 4 displays the absolute and relative frequency distribution for injury according to moment of injury (during training or competition). A significantly greater occurrence of injuries was found during training in comparison to during competition. No significant differences were found among the personal and training variables in relation to the moment of injury.

**Table 4:** Absolute and relative distribution of age, body mass, height and body mass index (BMI) according to moment of injury

**Variables**	**Characteristic**	**Moment of injury**	
		**Training**	**Competition**
**Age**	**Younger**^**1**^	31 (86.1)*	5 (13.9)
	**Older**^**2**^	62 (70.4)*	26 (29.6)
**Body mass**	**Lighter**^**3**^	44 (81.5)*	10 (18.5)
	**Heavier**^**4**^	49 (70.0)*	21 (30.0)
**Height**	**Short**^**5**^	46 (86.8)*	7 (13.2)
	**Tall**^**6**^	47 (66.2)*	24 (33.8)
**BMI**	**Less BMI**^**7**^	42 (77.8)*	12 (22.2)
	**More BMI**^**8**^	51 (72.8)*	19 (27.2)

^*1*^*cutoff point of lower bound of confidence interval (CI) (< 15.29 years) obtained from participants for age variable;*^*2*^*cutoff point for upper bound of CI (≥15.29 years) obtained from participants for age variable;*^*3*^*cutoff point of lower bound of CI (< 64.28 kg) obtained from participants for body mass variable;*^*4*^*cutoff point for upper bound of CI (≥64.28 kg) obtained from participants for body mass variable;*^*5*^*cutoff point of lower bound of CI (< 1.72 m) obtained from participants for height variable;*^*6*^*cutoff point for upper bound of CI (≥1.72 m) obtained from participants for height variable;*^*7*^*cutoff point of lower bound of CI (< 21.28 kg · m*^*-2*^*) obtained from participants for BMI variable;*^*8*^*cutoff point for upper bound of CI (≥21.28 kg · m*^*-2*^) obtained from participants for BMI variable; *Significant difference in relation competition (p < 0.05).

## Discussion

The investigation into injuries in young volleyball players demonstrates that the frequency of injuries was nearly 20% in the present study. Greater age, weight, height, BMI and duration of training values were associated with the occurrence of injuries. The most affected anatomic site was the ankle/foot complex among older, heavier, taller athletes and those with a greater BMI and longer duration of training. The direct contact and contactless mechanisms were the main causes of injury and the training phase was the moment in which more injuries were reported.

The rate of injury was 0.23 per athlete. Both Knowles *et al*. [5] and Powell *et al.*[19] report a risk of 0.14 injuries in young athletes. Despite the considerable frequency, young athletes appear to have a lesser chance of suffering injuries in comparison to high-performance athletes, which may be related to the greater physical demands in the latter group [20]. However, comparisons between studies of this nature are hampered by methodological differences, such as the definition of injury, the population studied and the manner in which the data are presented [10,21,22].

The rate of injury among injured athletes was 1.19. The risk of the occurrence of a new ankle ligament injury among volleyball players can be as much as fourfold higher than among those with no previous ankle injuries. Moreover, 50% of injured players may suffer further injury to the same site in the first six to 12 months following an injury. This risk of recurrence may be explained by the fact the tissue requires approximately six months to heal adequately and for muscle strength to completely recover. Moreover, the compromised proprioceptive function also increases the chances of further injury in ankle joint [23].

Aagaard and Jorgensen [19] conducted a study during the 1993–1994 season Danish to verify the occurrence of injuries in elite volleyball players through a questionnaire and found that 79 injuries were reported from a total of 70 female players and 98 injuries in 67 male players. While, Augustsson et al. [24], also by means of a questionnaire, observed 121 injuries in 82 elite volleyball players Swedish during the 2002–2003 season.

Powell et al. [19] conducted a study whose objective was to characterize the risk of injury in 10 popular sports in high school and found that the rate of injury per 1000 hours of exposure is greater in soccer (8.1), followed by basketball (4.8), and while the lowest rate of injury was observed in volleyball players (1.7). However, the authors concluded that the variation in the rate of injury depends on multiple factors such as the inherent risk of injury due to the nature of the game and the activities carried out by the players, beyond the time of sports practice and competition level.

Injuries occurred more among older, heavier, taller youths and those with a greater BMI and longer duration of training. Regarding age, a number of studies [25-27] report that older adolescents (16 to 19 years) have a greater chance of suffering injuries in comparison to younger adolescents (11 to 15 years), which may be related to the longer time practicing the sport and a more complex training regimen. Moreover, youths (especially older adolescents) are susceptible to sports injuries due to hormonal factors, such as the release of androgens, which is characteristic of this age group and generates an increase in muscle mass, speed and strength as well as a temporary decline in coordination, thereby leading to a greater predisposition to musculoskeletal injury during the practice of sports [28].

According to Frasson *et al.*[29] volleyball players need to be tall and physically strong in order to perform jumps, blocks and other motions related to this modality with efficacy and precision. The impact generated by these moves among taller, heavier players is potentially absorbed more by the joints and soft tissues in comparison to players with lower body mass, height and BMI values, leading to a greater chance of injury in the former group [20].

In the present study, the duration of training was also associated with the occurrence of injury. This finding corroborates that of a previous study in which a longer exposure time led to a greater chance of injury [5]. Exposure in hours has also been the focus of analysis in different studies [5,8], but this aspect was not investigated in the present study.

The ankle/foot complex was the most affected anatomic site among the participants in the present study. There is evidence that the ankle is quite vulnerable to injury in volleyball [8,27,30]. Ankle injuries occur most often as a consequence of the dynamic activities of the game, such as during landing, especially in the actions of stopping or blocking an attack, when the player’s foot may come into contact with the opponent, predisposing the individual to sprains, especially sprained ligaments [8]. The ligaments of this joint are more elastic in adolescents, which may also contribute toward the greater occurrence of injuries [25].

Although there were no significant differences in relation to the other anatomic sites, the wrist/hand complex exhibited a high frequency of injuries, independently of personal and training characteristics. This finding may be explained by contact with the ball, especially during blocking [31].

Direct contact was the most reported injury mechanism in the present study. Bahr and Bahr [23] and Labella [32] report that approximately 89% of volleyball injuries occur at the net during attacks or blocking, when the athletes land on the feet of other players. In this situation, the instability of the joint and accentuated inversion trigger injuries, especially in the anterior talofibular ligament. According to Aagard and Jorgensen [21], volleyball players perform upward and forward jumps when attacking, rather than only upward, often leading to contact below the net, which also contributes to injuries induced by this mechanism.

Regardless of the individual and training characteristics of the players, the occurrence of injury was higher during training than during competition. This may be related to the much longer exposure time during training than during matches. In contrast, Agel *et al.*[8] report that volleyball injuries predominate during matches, mainly due to the extra motivation generated during a competition. Moreover, the risk of injuries during competitions may be more frequent due to the greater intensity of the game [8]. We believe this study, analysis of the data for the occurrence of injuries may have influenced the findings, since there was made an association of musculoskeletal affections with of the hours of exposure to the player.

Some limitations should be identified from this study. The questionnaire used to collect information on injuries in volleyball is strategically adapted a model, but despite the simplicity of your questions has not yet been validated in the literature, which may represent a limitation of the study. However, although not validated, Pastre et al. [14] showed an excellent agreement between the occurrence of injuries and reporting of those on an interval of long time with the using this type of questionnaire. Furthermore, the investigation did not analyze tendencies in relation to the time athletes spend away from the sport due to injury or the severity of injuries – factors which could be useful to assessing injury patterns. Another limiting factor to be considered is the fact that the analysis does not explore the occurrence of injury according to players position on the court, which can also be a factor associated with occurrence of injury.

The consideration of cutoff points based on confidence intervals for mean values in the analysis of possible causal factors of sports injuries provides an interesting element to the discussion on injuries and their causes, therefore it can be established preventive measures based on values of anthropometric variables that favor the risk of injury. Moreover, the follow up of the occurrence of injuries and the investigation of associations in different age groups, including beginners, also appears to be important, as studies indicate that the rate of injury increases progressively with age [33,34].

## Conclusion

It found low frequency of injury among the volleyball players in the present study. Greater age, weight, height, BMI and duration of training values were associated with the occurrence of injuries. The most affected anatomic site was the ankle/foot complex among older, heavier, taller athletes and those with a greater BMI and longer duration of training. The direct contact and contactless mechanisms were the main causes of injury and the training phase was the moment in which more injuries were reported.

The prevention of sports injuries in young people is important and therefore the significant impact of these injuries in the population deserves special attention. The data from this study suggest that future research should focus on the development, implementation and evaluation of strategies for injury prevention in young volleyball players, based on anthropometric characteristics and ages of the participants.

The increasing sport participation of youth and the sports injuries lead to a significant impact on public health. This study identifies a clear need to direct young people playing volleyball on injury prevention strategies to maintain high rates of participation in physical activity and to reduce the risk of future health problems.

## Abbreviations

BMI: Index of body mass; OR: Odds ratio; CI: Confidence interval.

## Competing interests

The authors declare that they have no competing interests.

## Authors’ contributions

FNB, FMV and CMP conceived of the study, participated in its design and coordination and helped to draft the manuscript. FNB, FMV, GYCT, LCMV, JNJ and CMP performed the statistical analysis and interpretation of data and prepared the draft manuscript. All authors participated in the design of the study and in critical review of the manuscript. All authors read and approved the final manuscript.

## Supplementary Material

Additional file 1 **Appendix.** Reported Condition Inquiry. Click here for file
