# Supplementary material for: Characteristics and contributing factors related to sports injuries in young volleyball players
Source: BMC Res Notes. 2013 Oct 14;6:415. doi: 10.1186/1756-0500-6-415 (PMC4015734; doi:10.1186/1756-0500-6-415)
Supplement: Additional file 1 — Appendix. Reported Condition Inquiry. [file 1756-0500-6-415-S1.pdf]

Additional file 1: Appendix. Reported Condition Inquiry

Identification: Gender: ( ) Male ( ) Female
Date of birth:
Height: Weight: BMI:
City:
Duration of Training: Hours of practice per week:

Have you changed your training routine at any time in the current season due to an injury suffered during the practice of the sport?
( ) Yes ( ) No

If you answered **Yes,**
Fill in the following chart on the characteristics of the injury/injuries (anatomic site, mechanism and moment of injury), with the respective numeric identification listed in the second chart.

Characteristics of Injury/Injuries

	**1st**	**2nd**	**3rd**	**4th**	**5th**
**A – Anatomic site**					
**B – Injury mechanism**					
**C – Moment of injury**					

Codes for Variables

**A – Anatomic site**	**B – Injury mechanism**	**C – Moment of injury**
1 – Shoulder	1 – Direct contact	1 – Training
2 – Arm	2 – Contactless	2 – Competition
3 – Elbow	3 – Overuse	
4 – Forearm		
5 – Wrist		
6 – Hand		
7 – Anterior thigh		
8 – Posterior thigh		
9 – Knee		
10 – Leg		
11 – Calf		
12 – Ankle		
13 – Foot		
14 – Thorax		
15 – Abdomen		
16 – Head		
17 – Cervical spine		
18 – Lumbar spine		
19 – Pelvic girdle		
20 – Other		

Comment: (B – annotate action involved)

OBS:____
____
____
